# Association between breakfast composition and abdominal obesity in the Swiss adult population eating breakfast regularly

**DOI:** 10.1186/s12966-018-0752-7

**Published:** 2018-11-20

**Authors:** Angeline Chatelan, Katia Castetbon, Jerome Pasquier, Chloe Allemann, Alexandre Zuber, Esther Camenzind-Frey, Christine Anne Zuberbuehler, Murielle Bochud

**Affiliations:** 1 Institute of Social and Preventive Medicine (IUMSP), Lausanne University Hospital (CHUV), Route de la Corniche 10, 1010 Lausanne, Switzerland; 2 Ecole de Santé Publique, Centre de Recherche en Epidémiologie, Biostatistique et Recherche Clinique, Université libre de Bruxelles, Route de Lennik 808, 1070 Bruxelles, Belgium; 3 Risk Assessment Division, Nutrimonitoring Sector, Federal Food Safety and Veterinary Office (FSVO), Schwarzenburgstrasse 155, 3003 Bern, Switzerland

**Keywords:** National nutrition survey, menuCH, Breakfast, Dietary pattern, Abdominal obesity, Visceral fat, Waist-to-hip ratio, Waist circumference, Waist-to-height ratio, Swiss adults

## Abstract

**Background:**

Evidence from experimental and observational studies is limited regarding the most favorable breakfast composition to prevent abdominal fat accumulation. We explored the association between breakfast composition (a posteriori derived dietary patterns) and abdominal obesity among regular breakfast eaters from a Swiss population-based sample.

**Methods:**

The cross-sectional survey assessed diet using two 24-h dietary recalls in a nationally representative sample of adults aged 18 to 75 years. We derived dietary patterns using principal component analysis based on the intake of 22 breakfast-specific food groups. All regular breakfast eaters were predicted an individual score for each identified pattern, and then classified into tertiles (T1, T2, T3). We defined abdominal obesity as waist-to-hip ratio (WHR) ≥ 0.9 in men and ≥ 0.85 in women. Logistic models were adjusted for sociodemographic characteristics, relevant nutrition- and health-related behaviors, and diet quality during the rest of the day.

**Results:**

Of the 2019 included survey participants, 1351 (67%) were regular breakfast eaters. Among them, we identified three breakfast types: 1) ‘traditional’ − white bread, butter, sweet spread, 2) ‘prudent’ − fruit, unprocessed and unsweetened cereal flakes, nuts/seeds, yogurt, and 3) ‘western’ – processed breakfast cereals, and milk. The ‘prudent’ breakfast was negatively associated with abdominal obesity. After full adjustment, including diet quality during the rest of the day, the association was weaker (T3 vs. T1: OR 0.72, 95% CI: 0.47 to 1.08). People taking a ‘prudent’ breakfast (in T3) had 1.2% lower WHR compared to people taking a breakfast distant from ‘prudent’ (in T1) (*P* = 0.02, fully adjusted model with continuous log-WHR). We found no association between ‘traditional’ or ‘western’ breakfasts and WHR (OR 1.00, 95% CI: 0.67 to 1.50 and OR 1.16, 95% CI: 0.79 to 1.71, respectively). Findings were in the same directions for the three breakfast types when defining obesity with waist circumference, waist-to-height ratio, or body mass index (≥ 30 kg/m^2^, for ‘prudent’ breakfast: OR 0.51, 95% CI: 0.31 to 0.85).

**Conclusions:**

Regular breakfast consumers had less abdominal obesity if their breakfast was composed of fruit, natural cereal flakes, nuts/seeds and yogurt. This association was partly explained by their healthier diet during the rest of the day.

**Trial registration:**

ISRCTN16778734.

**Electronic supplementary material:**

The online version of this article (10.1186/s12966-018-0752-7) contains supplementary material, which is available to authorized users.

## Background

The impact of breakfast on obesity and cardio-metabolic health is disputed [1–5]. Two distinct aspects need to be considered: 1) breakfast skipping, and 2) type of breakfast in terms of food and nutrient composition. Breakfast also needs to be examined in the context of eating patterns throughout the entire day [6] because its consumption and composition may be related to meals and snacks consumed at other times of the day [7]. Cross-sectional and cohort studies have consistently reported skipping breakfast to be associated with an increased body weight [8–10]. However, experimental evidence is lacking to substantiate these observations [1, 2, 11, 12]. Fewer studies have investigated the impact of breakfast composition on cardio-metabolic risk factors [13]. There is growing experimental evidence suggesting that consuming a breakfast rich in protein and fiber is associated with benefits in terms of weight management [2] and cardio-metabolic health [3].

While intervention-based research is instrumental in deciphering causality, most clinical trials are of restricted duration (max. a few months) [2, 3], and hence do not assess the long-term impact of selected dietary behaviors on health. In addition, experimental studies often have limited external validity; indeed, their conclusions may not be generalized to the general population. Clearly, further research on whether and how breakfast may influence metabolic health is needed [2–5]. In this context, population-based observational studies provide important complementary evidence because they assess breakfast consumption in real-life settings, in more diverse populations, larger sample sizes, and over longer duration in case of longitudinal design. A few cross-sectional studies investigated the association of breakfast composition with body composition especially abdominal obesity in adult populations [13–17]. Studies in Canada [16] and the United States (U.S.) [14, 15, 17] using nationally representative food consumption data showed that adults taking breakfast made of pre- or un-sweetened ready-to-eat cereals or cooked cereals had lower waist circumference (WC) [14, 15] and/or body mass index (BMI) [14–17]. Iqbal et al. [13] showed in both male and female German middle-aged adults that eating salty protein-based breakfast was associated with increased BMI and WC. None of these studies accounted for diet quality during the rest of the day, and most used breakfast skippers as the comparison group. Such comparison is, however, less relevant when investigating the optimal breakfast composition because, as stated previously, most observational studies found that breakfast skippers had increased body weight [7–10].

Switzerland is located in the center of Europe and surrounded by three countries with very different dietary habits: France, Germany and Italy [18–20]. This unique multicultural setting revealed major differences in the consumption of food groups across the three main linguistic regions of the country [21]. In that sense, Switzerland represents an interesting setting to study how various dietary patterns may associate with abdominal obesity. In this study, we explored whether breakfast composition (a posteriori derived dietary patterns) were associated with abdominal obesity in Swiss regular breakfast eaters, using cross-sectional data from the first national nutrition survey, menuCH.

## Methods

We followed the STROBE-nut recommendations for reporting (Additional file 1) [22].

### Design and study population

We analyzed data from the Swiss Nutrition Survey menuCH collected between January 2014 and February 2015 [21]. menuCH is a cross-sectional, nationwide, population-based survey among adults aged 18 to 75 years living in Switzerland [21]. Selection of participants was based on a stratified random sample of the national sampling frame for surveys [23]. Response rate was 38%: of the 5496 eligible people reachable by phone, 2086 participated in the survey [21]. Participants and non-participants had similar age and marital status, but participants were more frequently women and Swiss nationals [21]. menuCH details are available at: https://menuch.iumsp.ch.

### Dietary assessment

Food consumption assessment was based on multiple-pass 24-h dietary recalls (24HDR), using the validated software GloboDiet®, previously known as EPIC-Soft [24, 25]. Dietitians conducted two non-consecutive 24HDR per participant. The first 24HDR was face-to-face and the second by phone, two to six weeks later. Food intake could have been recorded on any day of the week. When possible, dietitians planned interviews with participants on two different weekdays (e.g. not both on Mondays). Special days (e.g. party, holiday, or traveling days) were not excluded from analyses because of high frequency (i.e., about a third of 24HDR). Each food item was then linked with the most appropriate item from an extended research version of the Swiss Food Composition Database [26] (data available for energy, macronutrients, and sodium). For more details on dietary assessment and estimation of misreporting, read [21].

### Breakfast definition

We considered as breakfast all foods and beverages (including water) consumed in the food consumption occasions labeled by participants as ‘pre-breakfast (wake-up time)’ and ‘breakfast’. Breakfast was defined as skipped if less than 100 kcal were consumed. This cut-off choice was mainly data driven as shown in Additional file 2, but also based on literature [6]. Survey participants also reported in a questionnaire which day they usually skipped breakfast in a standard week (Monday to Sunday). For further analyses, we took only regular breakfast eaters into consideration, i.e., those breakfasting in both 24HDR and reporting eating breakfast at least 5 days in a standard week. Agreement between 24HDR and questionnaire was good: 93% of participants who consumed a breakfast in both 24HDR also reported taking a breakfast regularly in the questionnaire.

### Food group intake

Two registered dietitians independently classified foods and beverages into 36 groups of interest according to their nutritional value per typical portion size and their classification in the national food-based dietary guidelines [27] (Additional file 3). We then selected only the 22 food groups whose mean breakfast intake (in g) was at least 5% of the total daily intake. For example, vegetables were excluded because the mean breakfast intake represented 1% of the daily intake. We modeled the usual breakfast intake for the selected food groups using Multiple Source Method (MSM, https://nugo.dife.de/msm) [28–30].

### Definition of breakfast composition

We derived dietary patterns using principal component analysis (PCA, more specifically factor analysis) based on the standardized usual intake for the 22 food groups. In accordance with the scree plot (Additional file 4), we kept three factors. We applied varimax rotation to ease the interpretation. The food groups with a factor loading higher than an absolute value of 0.2 were considered as significant contributors to the pattern. We labelled the dietary patterns based on the food groups positively and negatively correlated to the identified patterns. Each regular breakfast eater was predicted a factor score for each pattern and was then categorized into a tertile (T1, T2, T3). The participants in the third tertile (T3) ate breakfast whose content was the closest to the pattern. The applicability of the data to factor analysis was considered as acceptable based on Kayser-Meyer-Olkin and the Bartlett’s sphericity tests (respectively, 0.59 and *P* < 0.001) [31, 32].

### Outcome assessment

We assessed abdominal obesity based on waist-to-hip ratio (WHR, ≥ 0.9 for men, 0.85 for women) [33]. To compare with literature and test whether our findings were dependent on the choice of the anthropometric parameters, we also used WC (i.e., obesity if WC >  90 cm for men, 84 cm for women) [33], waist-to-height ratio (WHtR, ≥ 0.5) [34–36], and BMI (≥ 30 kg/m^2^) [33]. Dietitians were extensively trained to measure body weight, height, waist and hip circumferences following an international protocol [37]. For waist and hip circumferences, we calculated the mean of the three consecutive measurements taken to the nearest 0.1 cm using a Gulick I unstretchable tape, equipped with a dynamometer (North Coast Medical, CA, USA).

### Covariates

We calculated total energy and nutrient intake (including alcohol) per recall day, but also at breakfast and during the rest of the day. We computed the mean nutrient intake out of the two days. The intakes of fiber, saturated fat, and sodium during the rest of the day were chosen as proxies for diet quality outside breakfast. We also estimated diet quality outside breakfast creating a nutritional score with six food components, selected from the 2010 Alternate Healthy Heating Index [38]: vegetables, fruit, whole grain, sugar sweetened drinks and fruit juice, nuts and legumes, and red and processed meat. More details about the scoring method and cut-offs are available in the Additional file 5.

Physical activity was assessed with the short-form International Physical Activity Questionnaire (IPAQ, six questions) [39, 40]. Data were converted into Metabolic Equivalent of Task (MET) minutes per week [41]. Information about education (university degree: yes/no), food literacy (knowing the existence of the Swiss Food Pyramid: yes/no), smoking (never/past/current), nationality (Swiss/non-Swiss), household status (alone/couple with children/couple without children) were assessed by questionnaire. The season was defined according to the date of the first 24HDR when anthropometric measurements were taken (April 15th to October 14th: warm/October 15th to April 14th: cold). Finally, we considered linguistic regions based on survey participants’ home address (German/French/Italian-speaking regions).

### Statistical analyses

We imputed missing data from the six IPAQ questions (between 1 and 16% of missing value for a single question) to passively calculate MET-min per week using multiple imputations by predictive mean matching through a Markov chain Monte Carlo method. We used multiple regressions accounting for sex, age, physical activity, and height to test the differences between T1 and T3 in food and nutrient intakes at breakfast and during the rest of the day. To assess the association between breakfast composition and abdominal obesity, we computed multiple logistic regressions using abdominal obesity assessed with WHR (WC, WHtR or BMI, respectively) as the binary outcome variable and the tertiles of breakfast type as exposure variables. For sensitivity analyses, we stratified by sex. We also applied multiple linear regression models using log-WHR as the outcome variable. The statistical significance of the differences in odds ratios between the three tertiles of each breakfast type was assessed using a Wald test. In addition, we estimated a *P*-value for trend based on a model considering the tertiles as a continuous exposure variable. We carried out all statistical analyses using STATA version 14 (Stata Corp., College Station, TX, USA).

## Results

Of the 2086 original survey participants [21], we excluded 67 of them (3%): 34 for missing waist and hip circumference measurements (i.e., 27 for pregnancy or lactation, 6 for handicap and 1 for refusal), 29 for missing second 24HDR, 4 for incomplete questionnaire on sociodemographic data and usual breakfast skipping days. Table 1 summarizes the characteristics of the included 2019 survey participants (46% of men). About one quarter of the survey sample presented a WHR above current recommendations and 13% were obese according to BMI measurement.

**Table 1:** Description of survey participants, by breakfasting regularity, and by breakfast type (by tertile in regular breakfast eaters)

Characteristics	All survey participants		Occasional breakfast eaters		Regular breakfast eaters^e^		‘Traditional’ – Pattern 1			‘Prudent’ – Pattern 2			‘Western’ – Pattern 3		
							T1^f^	T2	T3	T1	T2	T3	T1	T2	T3
*N* of participants	2019 (*100%*)		668 (33%)		1351 (67%)		451	450	450	451	450	450	451	450	450
Sex, *n (%)*															
Male	926	46	353	53	573	42	37	38	53	43	44	40	37	43	47
Female	1093	54	315	47	778	58	63	62	47	57	56	60	63	57	53
Age*, n (%)*															
18–34 years old	541	27	238	36	303	22	28	23	17	32	18	18	20	17	30
35–49 years old	588	29	205	31	383	28	30	29	26	31	27	27	26	29	29
50–64 years old	554	27	167	25	387	29	29	28	29	23	32	31	31	30	25
65–75 years old	336	17	58	9	278	21	13	20	29	14	24	24	22	23	16
Education: Highest degree, *n (%)*															
Secondary (e.g. apprenticeship and below)	1042	52	363	54	679	50	48	49	54	52	53	45	49	54	48
Tertiary (e.g. high technical school, university)	977	48	305	46	672	50	52	51	46	48	47	55	51	46	52
Total energy intake, *mean (±SD)*^a^															
Mean of two 24-h dietary recalls (*in kcal*)	2175	710	2092	740	2217	690	2110	2074	2467	2182	2243	2225	2106	2171	2373
Breakfast energy intake, *mean (±SD)*															
Mean of two 24-h dietary recalls (*in kcal*)	381	261	181	192	479	232	386	412	639	420	499	519	451	440	546
Self-reported physical activity, *mean (±SD)*															
MET-min per week (from IPAQ)	3761	3287	3816	3404	3730	3220	3580	3547	3974	3473	3960	3665	3751	3680	3654
Abdominal obesity assessment, *n (%)*															
Waist-to-hip ratio: ≥ 0.9 (♂); ≥ 0.85 (♀)^b,c^	546	27	191	29	355	26	21	27	31	24	32	24	25	29	25
Waist circumference: > 90 cm (♂); > 84 cm (♀)^b^	701	35	237	35	464	34	30	35	38	30	42	31	36	37	30
Waist-to-height ratio: ≥ 0.5 (♂, ♀)^d^	799	40	285	43	514	38	32	39	43	35	46	34	38	42	34
Body mass index: ≥ 30 kg/m^2^ (♂, ♀)^b^	255	13	114	17	141	10	9	11	11	12	12	7	10	10	11

^*a*^ *Mean daily energy intake was 2511 and 1891 kcal among all male and female survey participants, and 2574 and 1953 kcal among male and female regular breakfast eaters, respectively*

^*b*^*Cut-offs from the World Health Organization* [33]

^*c*^ *The proportions of men and women above the cut-off for waist-to-hip ratio was 44 and 13% among all survey participants, and 45 and 12% among regular breakfast eaters, respectively*

^*d*^*Cut-off suggested by Schneider* [34]*, Browning* [35] *and Ashwell* [36] et al.

^*e*^ *Regular breakfast eaters were defined as survey participants who took a breakfast of at least 100 kcal in both of their 24-h dietary recalls and reported eating breakfast at least 5 days in a usual week*

*Breakfast energy intake was 554 and 424 kcal among male and female regular breakfast eaters, respectively*

^*f*^ *In this second part of the table, only proportions, respectively, means for continuous variables, are presented in the 1351 regular breakfast eaters*

Sixty-seven per cent of survey participants (*N* = 1351) were regular breakfast eaters. Their mean daily energy intake, estimated out of two 24HDR, was 2217 kcal, respectively 2574 kcal for men and 1953 for women (Table 1). Mean (±SD) breakfast energy intake was 479 kcal (± 232), i.e., 554 and 424 kcal for men and women, respectively. The proportions of regular breakfast eaters with a WHR above the recommended cut-off were 26%; 45 and 12% in men and women, respectively.

After adjustment for sex, age, physical activity, total energy intake, education, food literacy, smoking, nationality, household status, season, and linguistic region, the odds of having an increased WHR were 1.6 times larger for occasional than for regular breakfast eaters (OR 1.59, 95% CI: 1.21 to 2.08, Additional file 6).

**Fig. 1:**
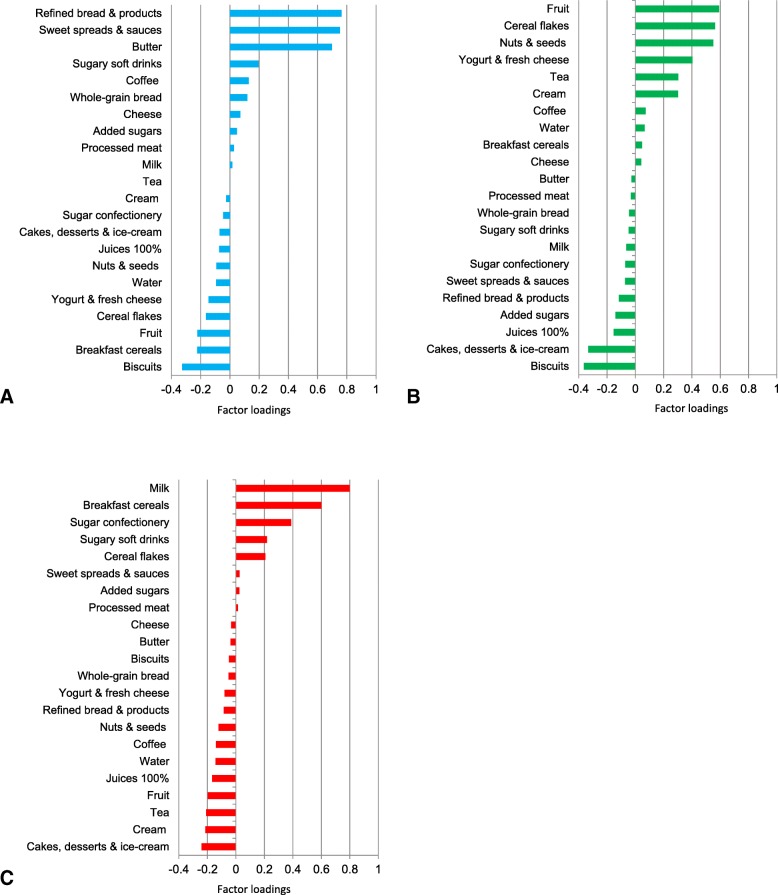
Breakfast dietary patterns. *Factor loadings for the three breakfast dietary patterns derived from for 22 food groups (y-axis). Pattern 1 (****a****) was called ‘Traditional’ (refined bread, butter, and sweet spread), Pattern 2 (****b****) ‘Prudent’ (‘Birchermuesli’), and Pattern 3 (****c****) ‘Western’ (processed breakfast cereals and milk)*

From PCA, three main dietary patterns emerged among the 1351 regular breakfast eaters (Fig. 1): 1) ‘traditional’ breakfast, rich in refined bread and bread products, butter and sweet spread (e.g. jam, honey), 2) ‘prudent’ breakfast, made of fruit, unprocessed and unsweetened cereal flakes, nuts/seeds and yogurt, which are typical ingredients of the Swiss recipe of ‘Birchermuesli’, and 3) ‘western’ breakfast, rich in processed and pre-sweetened breakfast cereals, milk, sugar confectionary and sugary soft drinks, including fruit nectars made of fruit juice, sugar and water. The cumulative percentage of explained variance was 26% (Additional file 4).

Table 1 shows that people adhering the most to the ‘traditional’ breakfast were rather older men with increased abdominal fat. Older and more educated people preferred the ‘prudent’ breakfast while younger men favored the ‘western’ breakfast. More details about participants’ characteristics by breakfast type can be found in Additional file 7.

Additional file 8 describes the nutrient intakes at breakfast by breakfast type. Succinctly, the ‘traditional’ breakfast was the richest in saturated fat and sodium. The ‘prudent’ breakfast had the highest fiber content. The median fiber intake among participants in T3 was more than doubled compared to those in T1 (6.7 g vs 2.8 g, + 3.9 g). After adjustment for sex, age, physical activity, and measured height, this difference was reduced to + 0.2 g (Additional file 8) but remains significant (*P* < 0.001). Additional files 9 and 10 show the differences in nutrient and food intakes between T1 and T3 for the rest of the day. In brief, for the ‘prudent’ breakfast, people classified in T3 scored significantly higher in the six-food-component nutritional score (+ 3%) compared to people in T1. For the ‘western’ breakfast, sugars intake were higher in people classified in T3 than those in T1. The former (T3) scored 4% below the latter (T1) in the six-food-component nutritional score.

After full adjustment for potential confounding factors, including diet quality for the rest of the day, the ‘traditional’ and ‘western’ breakfasts were not associated with elevated WHR (OR 1.00 for T3 vs. T1, 95% CI: 0.67 to 1.50, and OR 1.16, 95% CI: 0.79 to 1.71, respectively, Table 2). The ‘prudent’ breakfast was negatively associated with abdominal obesity. Participants with highest factor score (T3) for the ‘prudent’ pattern were less likely to have abdominal obesity than those with lowest factor score (T1). After adjustment for diet quality during the rest of the day (Model 4), the association became weaker and non-significant (T3 vs. T1: OR 0.72, 95% CI: 0.47 to 1.08, *P for trend* = 0.10). When WHR was analyzed continuously, a shift from T1 to T3 was associated with a significant difference of − 0.012 in log-WHR (Additional file 11). This corresponds to 1.2% lower WHR (95% CI, − 0.2% to − 2.2%). In sensitivity analyses, stratification by sex did not influence the results: odds ratios remained similar in both sexes for all three patterns (*data not shown*).

**Table 2:** Association between breakfast type and abdominal obesity (WHR ≥ 0.9 (♂); ≥ 0.85 (♀), *N* = 1351)

	Breakfast composition							
	T1	T2			T3			
	OR	OR	95% CI		OR	95% CI		*P*-Value for trend ^2^
‘Traditional’ – Refined bread, butter and sweet spread								
Crude	1 (ref)	1.40	1.03	1.90	1.72	1.27	2.32	< 0.001**
Model 1 *(sex + age)*	1 (ref)	1.25	0.87	1.80	0.89	0.61	1.28	0.45
Model 2 *(sex + age + physical activity + total energy intake)* ^*1*^	1 (ref)	1.31	0.91	1.90	0.93	0.64	1.36	0.67
Model 3 *(11 covariates)* ^*1*^	1 (ref)	1.39	0.95	2.03	1.00	0.68	1.48	0.95
Model 4 *(16 covariates, including diet quality during the rest of the day – nutrient + food-based approach)* ^*2*^	1 (ref)	1.32	0.90	1.93	1.00	0.67	1.50	0.99
‘Prudent’ – Fruit, unprocessed and unsweetened cereal flakes, nuts/seeds and yogurt								
Crude	1 (ref)	1.48	1.10	1.99	0.99	0.73	1.35	0.96
Model 1 *(sex + age)*	1 (ref)	0.98	0.68	1.40	0.60	0.41	0.87	0.006*
Model 2 *(sex + age + physical activity + total energy intake)* ^*1*^	1 (ref)	1.00	0.70	1.44	0.59	0.40	0.86	0.005*
Model 3 *(11 covariates)* ^*1*^	1 (ref)	1.01	0.70	1.47	0.60	0.41	0.90	0.011*
Model 4 *(16 covariates, including diet quality during the rest of the day – nutrient + food-based approach)* ^*2*^	1 (ref)	1.09	0.74	1.59	0.72	0.47	1.08	0.10
‘Western’ – Processed breakfast cereals and milk								
Crude	1 (ref)	1.24	0.93	1.67	1.00	0.74	1.36	0.98
Model 1 *(sex + age)*	1 (ref)	1.12	0.79	1.58	1.07	0.74	1.53	0.71
Model 2 *(sex + age + physical activity + total energy intake)* ^*1*^	1 (ref)	1.14	0.80	1.62	1.09	0.75	1.57	0.63
Model 3 *(11 covariates)* ^*1*^	1 (ref)	1.18	0.83	1.70	1.21	0.83	1.77	0.32
Model 4 *(16 covariates, including diet quality during the rest of the day – nutrient + food-based approach)* ^*2*^	1 (ref)	1.10	0.76	1.58	1.16	0.79	1.71	0.45

^1^ *Sex, age (continuous), physical activity (MET-min per week, continuous, imputed), total energy intake (mean out of two 24-h dietary recalls), alcohol intake (mean intake out of two 24-h dietary recalls), education (university degree*

*yes/no), food literacy (knowing about the Swiss Food Pyramid: yes/no), smoking (never/past/current), nationality (Swiss/non-Swiss), household status (alone/couple with children/couple without children), season of the first 24-h dietary recall (cold/warm), linguistic region (German/French/Italian)*

^2^ *Idem plus diet quality during the rest of the day (outside breakfast) considering dietary fiber, saturated fat, sodium, and the six-food-component nutritional score (mean intake out of two 24-h dietary recalls)*

^3^ Differences were assessed using multiple logistic regressions (* P ≤ 0.05, ** P ≤ 0.001)

**Fig. 2:**
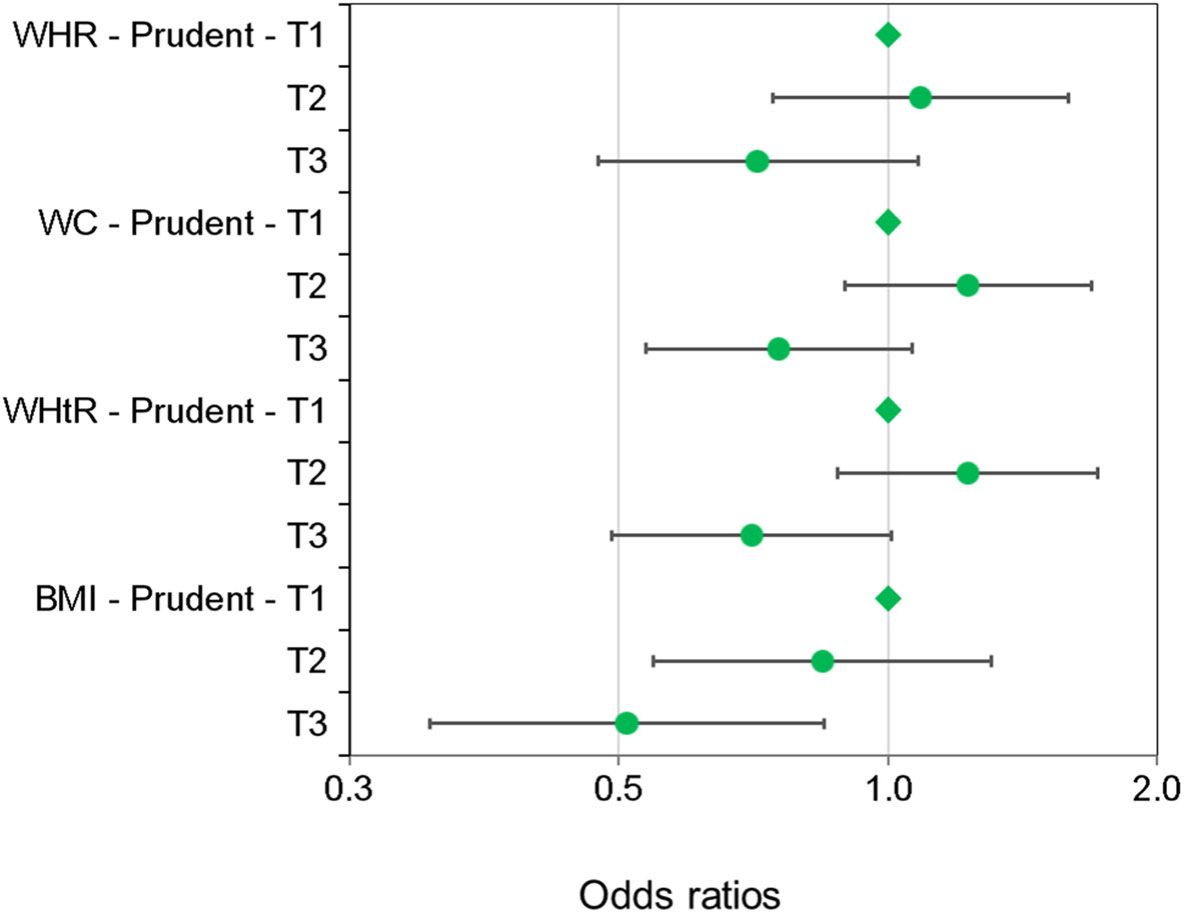
Association between the ‘prudent’ breakfast and four obesity anthropometric parameters. *Odds ratios between the ‘prudent’ breakfast (tertiles 1 to 3: T3 being closely associated with the pattern) and abdominal obesity (waist-to-hip ratio (WHR): ≥ 0.9 (♂); ≥ 0.85 (♀); waist circumference (WC): > 90 cm (♂); > 84 cm (♀), waist-to-height ratio (WHtR): ≥ 0.5 (♂, ♀), body mass index (BMI): ≥ 30 kg/m2 (♂, ♀), N = 1351). The logistic models were adjusted for sex, age, physical activity, total energy intake, alcohol intake, education, food literacy, smoking, nationality, household status, season of the first 24-h dietary recall, linguistic region, diet quality during the rest of the day (outside breakfast)*

Figure 2 compares the odds ratios between the ‘prudent’ breakfast and abdominal obesity assessed with the three other parameters (i.e., WC, WHtR or BMI) using the fully adjusted models (see Additional file 12 for the ‘traditional’ and ‘western’ patterns). We observed a significant negative association between the ‘prudent’ breakfast and BMI (OR 0.51, 95% CI: 0.31 to 0.85). When considering elevated WC and WHtR as outcomes, the associations were in the same direction although the CIs contained the null value.

## Discussion

We found out that consuming a ‘prudent’ breakfast composed of fruit, unprocessed and unsweetened cereal flakes, nuts/seeds and yogurt (typical Swiss recipe of ‘Birchermuesli’) was associated with less abdominal obesity in Swiss regular breakfast eaters. The association was partly due to higher overall diet quality in these people. Our finding is in line with results from a recent meta-analysis of 13 observational studies regarding overall dietary patterns also derived by PCA [42]. The highest category of ‘healthy/prudent’ pattern (with high-factor loadings in fruits, vegetables, and whole grain) was associated with reduced risk of central obesity compared with the lowest category (pooled OR 0.81, 95% CI: 0.66 to 0.96, I^2^ = 69.8) [42].

### High-fiber breakfast

In our research, all three breakfast types derived by PCA were rich in carbohydrates. Only the ‘prudent’ pattern ‘Birchermuesli’ had the particularity to be also rich in fiber coming from whole grain cereals, fruit and nuts/seeds, even though the higher fiber intake in T3 was partly confounded by differences in sex, age, physical activity, and height of the people in T3 compared to people in T1 (Additional file 8). The few randomized controlled trials that tested the effect of breakfast composition on cardio-metabolic health have also suggested that eating a high fiber breakfast might be the most protective strategy [2, 3]. Among those trials, one is particularly interesting for its relative long-term intervention. Adamsson et al. [43] demonstrated in 79 regular breakfast eaters that a normocaloric whole-grain cereal-based breakfast, which was very similar to our ‘prudent’ pattern, could reduce the sagittal abdominal diameter by 0.6 cm (*P* = 0.034). The authors also showed a reduction in circulating plasma inflammation markers within the three-month intervention. In our findings, Swiss regulars breakfast eaters taking a ‘prudent’ breakfast (in T3) had 1.2% lower WHR compared to those taking a breakfast distant from this pattern (in T1) (Additional file 11). Given the mean WHR at 0.829 in our sample and supposing the mean hip circumference staying constant at 100.1 cm, this would correspond to a mean difference of − 1 cm in WC.

The biological mechanisms behind the potential protective effect of consuming a breakfast rich in viscous and cereal fibers could be multiple. On the one hand, low-glycemic index carbohydrates, such as those in whole grain cereals, could lessen postprandial glucose response, limiting thus insulin production [3, 44–46]. On the other hand, dietary fiber may lower the release of free fatty acids from adipose tissues that cause insulin resistance. In turn, the diminution of insulin resistance reduces the production of pro-inflammatory mediators, and in fine, abdominal fat [3, 44, 47, 48]. Mediation mechanisms through the microbiota are likely to exist, stressing again the importance of dietary fiber for cardio-metabolic health [48].

### Breakfast composition in other population-based studies

Contrary to previous publications in North America, we did not detect an association between eating breakfast cereals (i.e., ‘western’ pattern) and abdominal obesity. In the U.S. National Health and Nutrition Examination Survey (NHANES) 1988–1994, eating ready-to-eat or cooked cereals, or quick breads was associated with significantly lower BMI compared to skipping breakfast or eating meats and/or eggs for breakfast [17]. Similarly, in young adults aged 20–39 years from the NHANES 1999–2006, breakfast including ready-to-eat cereals was associated with an improved cardio-metabolic risk profile [14]. O’Neil et al. also found in older adults that breakfasts composed of grains, pre- or un-sweetened ready-to-eat or cooked cereals, low-fat milk, and fruit were associated with lower BMI and WC than breakfast skipping [15]. The 2004 Canadian Community Health Survey also showed that mean BMI was significantly lower among consumers of ready-to-eat cereals at breakfast [16]. The fact that their comparison groups were breakfast skippers [15, 16], heterogeneous groups of ‘other breakfast’ consumers [14, 16], and/or groups with dietary patterns providing variable energy and nutrient intakes [15, 17] may explain the apparent inconsistency between North American and Swiss findings.

The other European study (in Germany) using PCA to derive breakfast composition from three 24HDR [13] found that the breakfast made of milk and breakfast cereals (undefined in terms of nutrient content) was not associated with increased or decreased WC, nor BMI. This ‘dairy & breakfast cereal pattern’ was, however, associated with a better multi-biomarker cardio-metabolic profile in men. The same article highlighted that the ‘processed food pattern’, composed of processed meat, cheese, vegetables, margarine, eggs, and bread, was positively associated with WC and BMI in both sexes. In Switzerland, a comparison between a high-fiber carbohydrate-based breakfast such as ‘Birchermuesli’ and a protein-based breakfast would have been interesting, since some evidence suggests that eating a protein-based breakfast may have beneficial effects too [2, 3]. Nevertheless, no protein-based breakfast emerged from the PCA as a main pattern, probably because this type of breakfast is less common than in Germany. Of note, the North American and German studies did not adjust for diet quality during the rest of the day.

While types of foods and beverages usually consumed at breakfast vary across countries, variations also exist in the contribution of breakfast to the daily energy intake. In our survey, breakfast brought 22% of total energy intake among regular breakfast eaters, and 18% among all survey participants, including occasional breakfast eaters. This proportion is slightly higher than in other western high-income countries: e.g., 14% in the Netherlands [49], 15% in Britain [50], 15% in the U.S. [15], 17% in France [51], or 16% in Spain [52]. This may represent different eating habits in the distribution of daily food consumption occasions, the proportion of breakfast skippers, and/or the definition of breakfast. Although breakfast accounts only for less than one fifth of the total energy intake across countries, understanding the impact of breakfast composition on health could complement the overall diet approach. This can also help define meal-based recommendations to assist populations in achieving the recommended daily intake [6, 53].

### Strengths and limitations

The present study has several strengths. First, we used data from a large, relatively representative sample of the Swiss population. Second, we focused on regular breakfast eaters to avoid comparison with breakfast skippers, who are known to have higher obesity prevalence in observational studies [1, 8–10] (Additional file 6). Third, specifically trained dietitians conducted the 24HDR using the internationally validated software GloboDiet®. In addition, we assessed the quality of 24HDR via several quality control procedures and underreporting was limited [21, 54]. Fourth, the same dietitian were also intensely trained to measure waist and hip circumferences. We tested inter-dietitian reproducibility during training sessions and organized two retraining sessions during the year of data collection. Intra-dietitian reproducibility was very high (Pearson’s correlation coefficients: *r* ≥ 0.99, *data not shown*). Fifth, we derived breakfast composition pattern based on the usual food intake modeled by MSM instead of using only the mean of two days**.** Sixth, we adjusted for most known confounders, including diet quality during the rest of the day. Seventh, our conclusions were independent of the choice of the anthropometric parameters we used as proxies of abdominal obesity.

The main limitation is the cross-sectional design. Thus, it is difficult to ascertain the temporal order of exposure and disease, essential for causal inference. Namely, people may have changed their diet for weight management. In addition, residual confounding may have biased the associations found between breakfast composition and abdominal obesity. Our results, however, open new hypotheses regarding the best choice for breakfast and complement the limited evidence from randomized controlled trials. An additional limitation is related to the method of PCA, which makes the comparison between groups/tertiles unintuitive. Indeed, breakfast eaters were not classified based on fixed food intake cut-offs, but their closeness or distance to a pattern. In other words, it is hard to picture the breakfast of people in the reference group (T1). Additionally, the three main identified dietary patterns explained only 26% of total variance. This indicates that breakfast patterns were complex and multiple in Switzerland. Thus, focusing only on three patterns explaining most variance reduces complexity but is imperfect. In our study, 27% of regular breakfast consumers were classified into none of the three T3 (Additional file 13), and 24% into more than one T3. These people respectively took other types of breakfast or foods overlapping several of the three selected patterns. We may also assume that some participants took one type of breakfast on the first recall day and another type on the second day. Currently, we know little about within-person variability in breakfast choice. In the U.S., Kant and Graubard showed that 17% of adults in NHANES 2005–2010 reported taking a breakfast in only one of the two 24HDR [55], and Sieger et al. [56] reported higher energy intake variability for snack and breakfast than for lunch and dinner. However, these references inform only about the variability in energy intake and not in food choices, which may be more limited at breakfast than for other meals, especially among regular breakfast eaters. Novel data mining techniques (e.g. maching learning algorithms) may help in defining more precisely the usual type of consumed meals [57, 58]. Furthermore, the inconsistent definitions of breakfast and breakfast skipping across studies and countries [6, 59] renders comparisons difficult. Finally, the method of 24HDR is sensitive to social desirability and recall bias, which may be important sources of under- or over-reporting in terms of food intake [60].

## Conclusions

Our study shows that a ‘prudent’ breakfast, based on fruit, unprocessed and unsweetened cereal flakes, nuts/seeds and yogurt, was associated with reduced abdominal obesity. This association was partly explained by a healthier diet during the rest of the day. Our findings need confirmation in other settings, such as in longitudinal studies, and, preferably, in long-term randomized controlled trials in free-living subjects.

## Additional files

Additional file 1: Completed STROBE-nut checklist. (DOCX 25 kb)

Additional file 2: Histogram of the variable energy intake consumed at breakfast per recall day using defined intervals of 50 kcal. (DOCX 17 kb)

Additional file 3: Description of foods and beverages included in the food groups used to derive dietary patterns. (DOCX 29 kb)

Additional file 4: Scree plot of eigenvalues and number of factors. (DOCX 16 kb)

Additional file 5: Calculation of the nutritional score used to assess food-based diet quality for the rest of the day. (DOCX 19 kb)

Additional file 6: Association between breakfast skipping and WHR. (DOCX 19 kb)

Additional file 7: Associations between covariates and breakfast composition patterns. (DOCX 22 kb)

Additional file 8: Assessment of differences in nutrient intakes at breakfast by breakfast type. (DOCX 25 kb)

Additional file 9: Assessment of differences in nutrient intakes for the rest of the day by breakfast type. (DOCX 25 kb)

Additional file 10: Assessment of differences in food intakes for the rest of the day by breakfast ype. (DOCX 25 kb)

Additional file 11: Association between breakfast type and WHR (continuous). (DOCX 21 kb)

Additional file 12: Association between the ‘prudent’ breakfast and four obesity anthropometric parameters. (DOCX 104 kb)

Additional file 13: Distribution of regular breakfast eaters in the three breakfast types. (DOCX 20 kb)

## Abbreviations

24HDR: 24-Hour Dietary Recalls
BMI: Body Mass Index
IPAQ: International Physical Activity Questionnaire
menuCH: First Swiss Nutrition Survey
MET: Metabolic Equivalent of Task
MSM: Multiple Source Method
NHANES: National Health and Nutrition Examination Survey
PCA: Principal Component Analysis
T: Tertile
U.S.: United States
WC: Waist Circumference
WHR: Waist-to-Hip ratio
WHtR: Waist-to-Height Ratio
