# Supplementary material for: Association between breakfast composition and abdominal obesity in the Swiss adult population eating breakfast regularly
Source: Int J Behav Nutr Phys Act. 2018 Nov 20;15:115. doi: 10.1186/s12966-018-0752-7 (PMC6247634; doi:10.1186/s12966-018-0752-7)
Supplement: Supplementary file 1 — Completed STROBE-nut checklist. (DOCX 25 kb) [file 12966_2018_752_MOESM1_ESM.docx]

Additional file 1. Completed STROBE-nut checklist.

| **Item** | No | Recommendation | Chapter |
| --- | --- | --- | --- |
| **Title and abstract** | nut-1 | State the dietary/nutritional assessment method(s) used in the title, abstract, or keywords | Abstract |
| **Introduction** Background/rationale | 2 | Explain the scientific background and rationale for the investigation being reported | Background |
| Objectives | 3 | State specific objectives, including any pre-specified hypotheses | Background (end) |
| **Methods**  Study design | 4 | Present key elements of study design early in the paper | Design and study population |
| Setting | 5 | Describe the setting, locations, and relevant dates, including periods of recruitment, exposure, follow-up, and data collection | Design and study population, Ref [21] |
|  | nut-5 | Describe any characteristics of study settings that might affect the dietary intake or nutritional status of the participants, if applicable | Design and study population, Ref [21] |
| Participants | 6 | (*a*) *Cohort study*—Give the eligibility criteria, and the sources and methods of selection of participants. Describe methods of follow-up  *Case-control study*—Give the eligibility criteria, and the sources and methods of case ascertainment and control selection. Give the rationale for the choice of cases and controls  *Cross-sectional study*—Give the eligibility criteria, and the sources and methods of selection of participants | NA  NA  Design and study population, Ref [21] |
|  |  | (*b*) *Cohort study*—For matched studies, give matching criteria and number of exposed and unexposed  *Case-control study*—For matched studies, give matching criteria and the number of controls per case | NA  NA |
|  | nut-6 | Report particular dietary, physiological, or nutritional characteristics that were considered when selecting the participants | NA |
| Variables | 7 | Clearly define all outcomes, exposures, predictors, potential confounders, and effect modifiers. Give diagnostic criteria, if applicable | Methods |
|  | nut-7.1 | Clearly define foods, food groups, nutrients, or other food components | Dietary assessment  Food group intake, Add. file 3 |
|  | nut-7.2 | When using dietary patterns or indices, describe the methods to obtain them and their nutritional properties | Definition of breakfast composition, Figure 1, Add. file 7 |
| Data sources/measurement | 8 | For each variable of interest, give sources of data and details of methods of assessment (measurement).  Describe comparability of assessment methods if there is more than one group | Methods, Ref [21]  NA |
|  | nut-8.1 | Describe the dietary assessment method(s), e.g., portion size estimation, number of days and items recorded, how it was developed and administered, and how quality was assured.  Report if and how supplement intake was assessed | Dietary assessment, Ref [21, 54]  NA |
|  | nut-8.2 | Describe and justify food composition data used.  Explain the procedure to match food composition with consumption data.  Describe the use of conversion factors, if applicable | Dietary assessment  Ref [21]  Dietary assessment, Ref [24-25] |
|  | nut-8.3 | Describe the nutrient requirements, recommendations, or dietary guidelines and the evaluation approach used to compare intake with the dietary reference values, if applicable | NA |
|  | nut-8.4 | When using nutritional biomarkers, additionally use the STROBE extension for molecular epidemiology (STROBE-me).  Report the type of biomarkers used and usefulness as dietary exposure markers | NA  NA |
|  | nut-8.5 | Describe the assessment of non-dietary data (e.g., nutritional status and influencing factors) and timing of the assessment of these variables in relation to dietary assessment | Covariates |
|  | nut-8.6 | Report on the validity of the dietary or nutritional assessment methods and any internal or external validation used in the study, if applicable | Dietary assessment, Strengths and limitations, Ref [24-25] |
| Bias | 9 | Describe any efforts to address potential sources of bias | Covariates, Strengths and limitations |
|  | nut-9 | Report how bias in dietary or nutritional assessment, e.g., misreporting, changes in habits as a result of being measured, and data imputation from other sources, was addressed | Covariates, Strengths and limitations, Ref [21] |
| Study size | 10 | Explain how the study size was arrived at | Ref [21] |
| Quantitative variables | 11 | Explain how quantitative variables were handled in the analyses. If applicable, describe which groupings were chosen and why | Covariates, Statistical analyses |
|  | nut-11 | Explain the categorization of dietary/nutritional data (e.g., use of n-tiles and handling of non-consumers) and the choice of reference category, if applicable | Definition of breakfast composition |

| Statistical methods | 12 | (*a*) Describe all statistical methods, including those used to control for confounding | Statistical analyses |
| --- | --- | --- | --- |
|  |  | (*b*) Describe any methods used to examine subgroups and interactions | Covariates, Statistical analyses, Add. file 5 |
|  |  | (*c*) Explain how missing data were addressed | Statistical analyses |
|  |  | (*d*) *Cohort study*—If applicable, explain how loss to follow-up was addressed  *Case-control study*—If applicable, explain how matching of cases and controls was addressed  *Cross-sectional study*—If applicable, describe analytical methods taking account of sampling strategy | NA  NA  NA |
|  |  | (*e*) Describe any sensitivity analyses | Statistical analyses |
|  | nut-12.1 | Describe any statistical method used to combine dietary or nutritional data, if applicable | Covariates, Add. file 5, 8 & 9 |
|  | nut-12.2 | Describe and justify the method for energy adjustments, intake modeling and use of weighting factors, if applicable | NA |
|  | nut-12.3 | Report any adjustments for measurement error, i.e., from a validity or calibration study | NA |
| **Results**  Participants | 13 | (a) Report numbers of individuals at each stage of study—eg numbers potentially eligible, examined for eligibility, confirmed eligible, included in the study, completing follow-up, and analyzed | Results, Table 1, Ref [21] |
|  |  | (b) Give reasons for non-participation at each stage | Design and study population, Results, Ref [21] |
|  |  | (c) Consider use of a flow diagram | Ref [21] |
|  | nut-13 | Report the number of individuals excluded based on missing, incomplete, or implausible dietary/nutritional data | Results, Ref [21] |
| Descriptive data | 14 | (a) Give characteristics of study participants (e.g. demographic, clinical, social) and information on exposures and potential confounders | Results, Table1 |
|  |  | (b) Indicate number of participants with missing data for each variable of interest | Results |
|  |  | (c) *Cohort study*—Summarize follow-up time (e.g., average and total amount) | NA |
|  | nut-14 | Give the distribution of participant characteristics across the exposure variables if applicable. Specify if food consumption of total population or consumers only were used to obtain results | Results, Table 1, Add. files 7, 8 & 9 |
| Outcome data | 15 | *Cohort study*—Report numbers of outcome events or summary measures over time | NA |
|  |  | *Case-control study—*Report numbers in each exposure category, or summary measures of exposure | NA |
|  |  | *Cross-sectional study—*Report numbers of outcome events or summary measures | Results, Table1 |
| Main results | 16 | (*a*) Give unadjusted estimates and, if applicable, confounder-adjusted estimates and their precision (eg, 95% confidence interval). Make clear which confounders were adjusted for and why they were included | Results, Table 2 |
|  |  | (*b*) Report category boundaries when continuous variables were categorized | Outcome assessment, Figure 2 |
|  |  | (*c*) If relevant, consider translating estimates of relative risk into absolute risk for a meaningful time period | Results |
|  | nut-16 | Specify if nutrient intakes are reported with or without inclusion of dietary supplement intake, if applicable | NA |
| Other analyses | 17 | Report other analyses done − eg analyses of subgroups and interactions, and sensitivity analyses | Results, Figure 2, Add. file 6 |
|  | nut-17 | Report any sensitivity analysis (e.g., exclusion of misreporters or outliers) and data imputation, if applicable | NA |
| **Discussion**  Key results | 18 | Summarize key results with reference to study objectives | Discussion |
| Limitations | 19 | Discuss limitations of the study, taking into account sources of potential bias or imprecision. Discuss both direction and magnitude of any potential bias | Strengths and limitations |
|  | nut-19 | Describe the main limitations of the data sources and assessment methods used and implications for the interpretation of the findings | Strengths and limitations |
| Interpretation | 20 | Give a cautious overall interpretation of results considering objectives, limitations, multiplicity of analyses, results from similar studies, and other relevant evidence | Conclusion |
|  | nut-20 | Report the nutritional relevance of the findings, given the complexity of diet or nutrition as an exposure | Discussion |
| Generalisability | 21 | Discuss the generalisability (external validity) of the study results | Background, Strengths and limitations |
| **Other information**  Funding | 22 | Give the source of funding and the role of the funders for the present study and, if applicable, for the original study on which the present article is based | Funding, Ref [21] |
|  | nut-22.1 | Describe the procedure for consent and study approval from ethics committee(s) | Ethics approval and consent to participate |
|  | nut 22.2 | Provide data collection tools and data as online material or explain how they can be accessed | Availability of data and material |
