# Supplementary material for: Association between breakfast composition and abdominal obesity in the Swiss adult population eating breakfast regularly
Source: Int J Behav Nutr Phys Act. 2018 Nov 20;15:115. doi: 10.1186/s12966-018-0752-7 (PMC6247634; doi:10.1186/s12966-018-0752-7)
Supplement: Supplementary file 2 — Histogram of the variable energy intake consumed at breakfast per recall day using defined intervals of 50 kcal. (DOCX 17 kb) [file 12966_2018_752_MOESM2_ESM.docx]

Additional file 2. Histogram of the variable energy intake consumed at breakfast per recall day using defined intervals of 50 kcal (N=2019).
