# Supplementary material for: Association between breakfast composition and abdominal obesity in the Swiss adult population eating breakfast regularly
Source: Int J Behav Nutr Phys Act. 2018 Nov 20;15:115. doi: 10.1186/s12966-018-0752-7 (PMC6247634; doi:10.1186/s12966-018-0752-7)
Supplement: Supplementary file 3 — Description of foods and beverages included in the food groups used to derive dietary patterns. (DOCX 29 kb) [file 12966_2018_752_MOESM3_ESM.docx]

| **Swiss Food Pyramid stage** | **Food groups** | **Foods classified in the group** | **Mean quantity**  **brought by breakfast**  ***(in g and % of daily intake)*** | |  |
| --- | --- | --- | --- | --- | --- |
|  | **Water** | All types of water (e.g. tap, mineral, still, carbonated), *essentially (99%) consumed without added sugars/artificial sweeteners.* | 149.8 | **13%*** |  |
| Non-caloric beverages | **Coffee** | All types of coffee (e.g. instant, from capsules), and coffee with milk or cream (e.g. cappuccino), *essentially (98%) consumed without added sugars/artificial sweeteners.* | 107.8 | **44%*** |  |
|  | **Tea** | All types of tea, herbal and fruit tea, *essentially (98%) consumed without added sugars/artificial sweeteners.* | 96.8 | **28%*** |  |
| Fruit & vegetables | Vegetables | All types of vegetables, green leaves, sprouts, mushrooms, seaweeds, sweet corn, snow peas, fresh green beans, and onions: *raw, cooked, dried, canned, in puree, pickled, in soups, in sauce (e.g. tomato sauce), on pizza and quiches, and in sandwiches.* Except: avocadoes, olives, herbs, vegetable juices, nor if contained in small amounts in salty snacks, bread, or sauces. | 1.9 | 1% |  |
|  | **Fruit** | All types of fruit: *raw, cooked, dried, in puree/compote, and in pies made essentially with fruit (e.g. apples in apple pies).* Except: fruit juices, fruit jams, candied fruit, nor if contained in small amounts in yogurts, cakes, ice-cream or other sweets. | 44.3 | **23%*** |  |
|  | Tubers | Unprocessed tubers (e.g. potatoes, sweet potatoes). | 0.2 | 1% |  |
|  | Tuber products | Potato products (e.g. French fries, rösti, mashed potatoes). | 0.2 | 2% |  |
|  | Whole-grain bread | All types of bread with more than 4.5g of dietary fibers per 100g (e.g. whole-wheat bread or bread rolls, rye bread) | 14.1 | **47%*** |  |
|  | Refined bread & bread products | All types of white or semi-white bread with less than 4.5g of dietary fibers per 100g (e.g. baguette, milk bread rolls, white toast, refined flat bread), croissants, and crisp bread (e.g. rice crackers, Swedish rolls). Except: stuffed croissants and sweet pastries (e.g. chocolate croissants). | 36.1 | **41%*** |  |
| Cereal products & potatoes | Pasta | Plain pasta (e.g. penne, spaghetti), schupfnudeln, spatzli, rice noodles, and stuffed pasta (e.g. ravioli, tortellini). | 0.1 | 0% |  |
|  | Rice | All types of rice. | 0.3 | 1% |  |
|  | Cereal flakes | Natural cereal flakes, oatmeal, flakes from birchermuesli and porridge, dried wheat germs, and natural cereal bran, *with or without dried fruit and/or nuts, without added sugars/artificial sweeteners nor major food processing.* | 5.3 | **84%*** |  |
|  | Breakfast cereals | Ready-to-eat and processed breakfast cereals (e.g. corn-flakes), and birchermuesli mixes, *highly processed and/or with added sugars/artificial sweeteners.* | 5.4 | **82%*** |  |

|  | Milk | Mammals’ milk, branded fermented milk drinks (e.g. bifidus), yogurt drink and buttermilk, *in liquid form, essentially (96%) without added sugars/artificial sweeteners.* | 77.8 | **62%*** |  |
| --- | --- | --- | --- | --- | --- |
|  | Yogurt & fresh cheese | Mammals’ yogurt, branded fermented milk and kefir, fresh cheese (e.g. petit suisse, quark, cottage cheese, ricotta), *in semi-solid form, mostly (67%) with added sugars/artificial sweeteners.* | 32.3 | **47%*** |  |
|  | Cheese | Mammals’ soft and hard cheese, spread cheese, processed/melted cheese. | 5.4 | **13%*** |  |
| Protein-based products | Red meat | - Fresh meat and offal from beef, veal, pork, lamb, mutton, horse, goat, rabbit, and wild red meat (e.g. venison/deer) | 0.2 | 1% |  |
|  | Poultry | - Fresh meat and offal from chicken, turkey, duck, goose, and ostrich. | 0.2 | 1% |  |
|  | Processed meat | Sausages, cold cuts, smoked or cured meat (e.g. ham, bacon, salami, corned beef, beef jerky, meat terrine), meat-based sauces (e.g. Bolognese sauce), meat-based spread (e.g. liver spread), and minced meat for burgers. | 1.6 | **5%*** |  |
|  | Fish & seafood | All types of fresh fish, seafood and snails, and processed fish and seafood products (e.g. fish in crumb, surimi). | 0.3 | 2% |  |
|  | Vegetable oils | Added vegetable oils (e.g. rapeseed oil, olive oil, sunflower oil). Except: coco fat used to cook. | 0.2 | 3% |  |
|  | Butter | Added butter to cook and spread on bread. | 5.6 | **58%*** |  |
|  | Cream | All types of mammals’ added creams. | 1.4 | **11%*** |  |
| Added fats & oils | Sauces rich in fats | Sauces rich in oil or in butter (e.g. mayonnaise, pesto, sauce café de Paris), other sauces rich in cream or other fats (e.g. carbonara, cocktail sauce, hummus, satay sauce), and coco milk. | 0.1 | 1% |  |
|  | Dressing sauces | All types of dressing sauces. | 0.0 | 0% |  |
|  | Nuts & seeds | Dried nuts and seeds (e.g. almonds, hazelnuts, coconut), olives, and avocadoes, *with or without salt/sugars.* | 1.6 | **16%*** |  |
|  | Added sugars | All types of beet or cane sugars, polyols. Except: stevia and artificial sweeteners. | 1.3 | **33%*** |  |
|  | Sweet spreads & sauces | Jams, jelly, honey, syrups, sweet sauces (e.g. caramel or chocolate sauce, maple syrup), chocolate spread, peanut butter, and sweet topping (e.g. icing). | 17.5 | **78%*** |  |
| Sweets, salty snacks & alcohol | Cakes, desserts & ice-cream | All types of sweet cakes, pies and tarts (e.g. brownies, lemon cakes, muffins), sweet pastries (e.g. waffles, chocolate croissants, doughnuts), desserts made with dairy products (e.g. pudding, chocolate mousse, tiramisu), ice-cream, sorbet, iced cakes. | 2.3 | **6%*** |  |
|  | Biscuits | All types of sweet and dried biscuits (e.g. shortbread, amaretto, Christmas biscuits, chocolate chip cookies, leckerli, meringue). | 1.0 | **12%*** |  |

|  | Sugar confectionery | All types of sweets and candies (e.g. marzipan, candied fruit, lollipop, marshmallows), pure chocolate, chocolate products and confections (e.g. filled chocolate, pralines, branded chocolate bars), sports cereal bars (e.g. energy bars), and sweet pop corn. | 0.8 | **6%*** |  |
| --- | --- | --- | --- | --- | --- |
|  | Salty snacks | Crisps, flips, salty popcorn, dried salty crackers (e.g. golden fish biscuits, sticks, pretzels), cocktail canapés, crostini/bruschetta, spring rolls, ham croissants, samosa, and sausage rolls. | 0.3 | 4% |  |
| Sweets, salty snacks & alcohol | Juices 100% | 100% fruit and vegetable juices (fresh or from concentrate), smoothie, fruit-schorle (i.e. fruit juices, often apple juice, mixed with water), *all without added sugars/artificial sweeteners.* | 31.4 | **39%*** |  |
|  | Sugary soft drinks | Sweetened soft drinks, sports drinks, energy drinks, fizzy drinks, ice tea, diluted syrup, drinks made with fruit juice and water (e.g. fruit nectars, lemonades), alcohol-free beers, *with added sugars.* | 13.9 | **11%*** |  |
|  | Beer | All types of alcoholic beers, apple or pear ciders, and shandy (e.g. beer with lemonade). | 0.0 | 0% |  |
|  | Wine | White and red wine, champagne, wine products (e.g. sangria, punch/bowle with alcohol), port, sherry, and vermouths. | 0.0 | 0% |  |

** Relevant for derivation of breakfast dietary patterns because ≤ 5% of the intake (in g) is brought by breakfast.*
