# Supplementary material for: Association between breakfast composition and abdominal obesity in the Swiss adult population eating breakfast regularly
Source: Int J Behav Nutr Phys Act. 2018 Nov 20;15:115. doi: 10.1186/s12966-018-0752-7 (PMC6247634; doi:10.1186/s12966-018-0752-7)
Supplement: Supplementary file 5 — Calculation of the nutritional score used to assess food-based diet quality for the rest of the day. (DOCX 19 kb) [file 12966_2018_752_MOESM5_ESM.docx]

**Additional file 5.** Components and scoring method of the nutritional score used to assess food-based diet quality for the rest of the day (i.e. without breakfast) based on the 2010 Alternate Healthy Eating Index^1,2^

| **Components** | **Criteria for minimum score (0)** | **Criteria for maximum score (10)** |
| --- | --- | --- |
| Vegetables, excluding potatoes (servings/day)^3^ | 0 | ≥ 5 |
| Fruit, excluding juice (servings/day)^4^ | 0 | ≥ 4 |
| Whole grains (g/day)^5^ |  |  |
| Women | 0 | ≥ 75 |
| Men | 0 | ≥ 90 |
| Sugar-sweetened beverage and fruit juice (servings/day)^6^ | ≥ 1 | 0 |
| Nuts, seeds, legumes, and tofu (servings/day)^7^ | 0 | ≥ 1 |
| Red and processed meat (servings/day)^8^ | ≥ 1.5 | 0 |

*^1^ Adapted from Chiuve et al. 2012.*

*^2^ Intermediate food intake was scored proportionately between the minimum score 0 and the maximum score 10.*

*^3^ One serving was equal to 118.3g of raw or cooked vegetables, 30g of dried vegetables or 250g of homemade vegetable soup.*

*^4^ One serving was equal to 118.3g of raw or cooked fruit or 30g of dried fruit.*

*^5^ Any grain food (e.g. rice, pasta, bread, breakfast cereals, etc.) with a carbohydrate-to-fiber ratio smaller than 10:1 was considered as whole grain.*

*^6^ One serving was equal to 226.8g.*

*^7^ One serving was equal to 28.4g.*

*^8^ One serving was equal to 113.4g of red meat or 42.5g of processed meat.*
