# Supplementary material for: Association between breakfast composition and abdominal obesity in the Swiss adult population eating breakfast regularly
Source: Int J Behav Nutr Phys Act. 2018 Nov 20;15:115. doi: 10.1186/s12966-018-0752-7 (PMC6247634; doi:10.1186/s12966-018-0752-7)
Supplement: Supplementary file 6 — Association between breakfast skipping and WHR. (DOCX 19 kb) [file 12966_2018_752_MOESM6_ESM.docx]

**Additional file 6.** Association between breakfast skipping and abdominal obesity (WHR ≥ 0.9 (♂); ≥ 0.85 (♀), N=2019).

|  | **Regular eaters**  **(N=1531)** |  | **Occasional eaters**  **(N=668)** | | |  | |
| --- | --- | --- | --- | --- | --- | --- | --- |
|  | **OR** |  | **OR** | **95% CI** | | **P-Value** |  |
| **Crude** | 1 (ref) |  | 1.12 | 0.91 | 1.38 | 0.27 |  |
| **Model 1** (sex + age) | 1 (ref) |  | 1.67 | 1.29 | 2.16 | <0.001** |  |
| **Model 2** *(11 covariates)* ^1^ | 1 (ref) |  | 1.59 | 1.21 | 2.08 | <0.001** |  |

*^1^ Sex, age (continuous), physical activity (MET-min per week, continuous, imputed), total energy intake (mean out of two 24-hour dietary recalls), education (university degree: yes / no), food literacy (knowing about the Swiss Food Pyramid: yes / no), smoking (never / past / current), nationality (Swiss / non-Swiss), household status (alone / couple with children / couple without children), season of the first 24-hour dietary recall (cold / warm), linguistic region (German / French / Italian).*

*^2^ Differences were assessed using multiple logistic regressions (** P ≤ 0.001).*
