# Supplementary material for: Association between breakfast composition and abdominal obesity in the Swiss adult population eating breakfast regularly
Source: Int J Behav Nutr Phys Act. 2018 Nov 20;15:115. doi: 10.1186/s12966-018-0752-7 (PMC6247634; doi:10.1186/s12966-018-0752-7)
Supplement: Supplementary file 7 — Associations between covariates and breakfast composition patterns. (DOCX 22 kb) [file 12966_2018_752_MOESM7_ESM.docx]

Additional file 7. Associations between all the covariates and breakfast composition patterns (N=1351).

| **Covariates** | **Association with the ‘traditional’ breakfast** ^1^ | | **Association with the ‘prudent’ breakfast** ^1^ | | **Association with the ‘western’ breakfast** ^1^ | |
| --- | --- | --- | --- | --- | --- | --- |
|  | **OR** | **P-Value** | **OR** | **P-Value** | **OR** | **P-Value** |
| **Sex**  Male | 1 (ref.) |  | 1 (ref.) |  | 1 (ref.) |  |
| Female | 0.597 | <0.001** | 1.097 | 0.361 | 0.728 | 0.002* |
| **Age**, *y* | 1.020 | <0.001** | 1.021 | <0.001** | 0.986 | <0.001** |
| **Physical activity**, *MET-min per week* | 1.00009 | <0.001** | 1.000003 | 0.877 | 1.00001 | 0.593 |
| **Total energy intake**, *kcal* | 1.0006 | <0.001** | 1.0001 | 0.349 | 1.0004 | <0.001** |
| **Education**  Secondary (e.g. apprenticeship and below) | 1 (ref.) |  | 1 (ref.) |  | 1 (ref.) |  |
| Tertiary (e.g. high technical school, university) | 0.816 | 0.042 * | 1.242 | 0.030* | 1.051 | 0.618 |
| **Food literacy**  No | 1 (ref.) |  | 1 (ref.) |  | 1 (ref.) |  |
| Yes | 0.797 | 0.081 | 1.054 | 0.681 | 0.912 | 0.479 |
| **Linguistic region**  German | 1 (ref.) |  | 1 (ref.) |  | 1 (ref.) |  |
| French | 1.064 | 0.595 | 0.486 | <0.001** | 0.708 | 0.003* |
| Italian | 0.557 | 0.001* | 0.191 | <0.001** | 0.764 | 0.102 |
| **Smoking**  Never | 1 (ref.) |  | 1 (ref.) |  | 1 (ref.) |  |
| Past | 1.002 | 0.988 | 1.097 | 0.435 | 0.794 | 0.052 |
| Current | 0.892 | 0.406 | 0.675 | 0.005* | 1.046 | 0.742 |
| **Season** of the first 24-hour dietary recall  Cold | 1 (ref.) |  | 1 (ref.) |  | 1 (ref.) |  |
| Warm | 0.997 | 0.976 | 0.884 | 0.217 | 0.783 | 0.015* |
| **Nationality**  Non-Swiss | 1 (ref.) |  | 1 (ref.) |  | 1 (ref.) |  |
| Swiss | 1.995 | <0.001** | 1.363 | 0.030* | 1.313 | 0.047* |
| **Household status**  Alone | 1 (ref.) |  | 1 (ref.) |  | 1 (ref.) |  |
| Couple with children | 1.016 | 0.882 | 0.589 | <0.001** | 1.037 | 0.735 |
| Couple without children | 0.877 | 0.435 | 0.574 | 0.001* | 1.667 | 0.003* |
| **Alcohol**, *g* | 1.004 | 0.229 | 0.998 | 0.407 | 0.993 | 0.016* |
| **Fiber intake during the rest of the day**, *g* | 0.993 | 0.347 | 1.042 | <0.001** | 1.001 | 0.870 |
| **Saturated fat intake during the rest of the day**, *g* | 1.014 | <0.001** | 1.004 | 0.345 | 1.013 | <0.001** |
| **Sodium intake during the rest of the day**, *g* | 1.076 | 0.092 | 0.901 | 0.015* | 1.124 | 0.006* |
| **6-food-component score during the rest of the day***, 0-60* | 0.978 | <0.001** | 1.048 | <0.001** | 0.976 | <0.001** |

*^1^ Assessed with univariate ordered logistic regression (T1, T2, T3, * P ≤ 0.05, ** P ≤ 0.001).*
