# Supplementary material for: Association between breakfast composition and abdominal obesity in the Swiss adult population eating breakfast regularly
Source: Int J Behav Nutr Phys Act. 2018 Nov 20;15:115. doi: 10.1186/s12966-018-0752-7 (PMC6247634; doi:10.1186/s12966-018-0752-7)
Supplement: Supplementary file 8 — Assessment of differences in nutrient intakes at breakfast by breakfast type. (DOCX 25 kb) [file 12966_2018_752_MOESM8_ESM.docx]

Additional file 8. Differences in nutrient intakes at breakfast (unadjusted plus adjusted medians of the mean intake out of two 24-hour dietary recalls) by breakfast type (Tertiles, T1 vs. T3, N=1351).

Unadjusted medians:

|  | ‘Traditional’ – Pattern 1 | | | | | ‘Prudent’ – Pattern 2 | | | | | ‘Western’ – Pattern 3 | | | | | |
| --- | --- | --- | --- | --- | --- | --- | --- | --- | --- | --- | --- | --- | --- | --- | --- | --- |
|  | **T1** | **T2** | **T3** | **T3 / T1 (%)** | **P-Value** ^1^ | **T1** | **T2** | **T3** | **T3 / T1 (%)** | **P-Value** ^1^ | **T1** | **T2** | **T3** | **T3 / T1 (%)** | **P-Value** ^1^ |  |
| Energy (kcal) | 350.4 | 387.3 | 595.8 | 170% | <0.001** | 381.6 | 446.6 | 482.4 | 126% | <0.001** | 396.4 | 407.1 | 493.8 | 125% | <0.001** |  |
| Protein (g) | 11.1 | 10.8 | 14.7 | 133% | <0.001** | 10.5 | 12.1 | 14.2 | 135% | <0.001** | 10.2 | 10.3 | 15.8 | 155% | <0.001** |  |
| Total carbohydrate (g) | 47.3 | 50.3 | 79.9 | 169% | <0.001** | 52.1 | 57.9 | 63.6 | 122% | <0.001** | 56.3 | 53.7 | 64.8 | 115% | <0.001** |  |
| Sugars (g) | 27.7 | 24.5 | 38.7 | 140% | <0.001** | 25.7 | 28.3 | 36.0 | 140% | <0.001** | 30.4 | 25.7 | 34.4 | 113% | 0.010* |  |
| Fiber (g) | 4.2 | 3.6 | 5.1 | 120% | 0.002* | 2.8 | 4.0 | 6.7 | 235% | <0.001** | 4.4 | 3.8 | 4.8 | 109% | 0.09 |  |
| Total Fat (g) | 11.0 | 13.1 | 22.0 | 200% | <0.001** | 13.8 | 16.1 | 15.2 | 111% | 0.08 | 13.7 | 14.2 | 16.5 | 121% | 0.001** |  |
| Saturated fat (g) | 4.6 | 6.4 | 11.0 | 238% | <0.001** | 6.6 | 7.8 | 6.1 | 93% | 0.28 | 6.2 | 6.6 | 7.7 | 123% | 0.001** |  |
| Sodium / Na (mg) | 172.6 | 354.9 | 594.5 | 344% | <0.001** | 356.8 | 446.4 | 258.1 | 72% | <0.001** | 333.2 | 376.2 | 359.5 | 108% | 0.30 |  |

*^1^ Differences between T1 and T3 were assessed using Wald tests on quantile regression coefficients (no adjustment, * P ≤ 0.05, ** P ≤ 0.001).*

Adjusted^1^ medians:

|  | ‘Traditional’ – Pattern 1 | | | | | ‘Prudent’ – Pattern 2 | | | | | ‘Western’ – Pattern 3 | | | | | |
| --- | --- | --- | --- | --- | --- | --- | --- | --- | --- | --- | --- | --- | --- | --- | --- | --- |
|  | **T1** | **T2** | **T3** | **T3 / T1 (%)** | **P-Value** ^2^ | **T1** | **T2** | **T3** | **T3 / T1 (%)** | **P-Value** ^2^ | **T1** | **T2** | **T3** | **T3 / T1 (%)** | **P-Value** ^2^ |  |
| Energy (kcal) | 433.0 | 434.4 | 457.1 | 106% | <0.001** | 438.5 | 443.3 | 440.9 | 101% | <0.001** | 433.1 | 442.6 | 447.0 | 103% | <0.001** |  |
| Protein (g) | 12.1 | 12.0 | 12.5 | 104% | <0.001** | 12.3 | 12.2 | 12.1 | 99% | <0.001** | 11.9 | 12.2 | 12.5 | 104% | <0.001** |  |
| Total carbohydrate (g) | 58.1 | 58.1 | 60.8 | 105% | <0.001** | 59.1 | 59.2 | 58.8 | 99.6% | <0.001** | 57.9 | 59.1 | 60.1 | 104% | 0.012* |  |
| Sugars (g) | 30.1 | 30.2 | 31.7 | 105% | <0.001** | 30.7 | 30.8 | 30.6 | 99.7% | <0.001** | 30.0 | 30.7 | 31.2 | 104% | 0.16 |  |
| Fiber (g) | 4.3 | 4.3 | 4.5 | 105% | 0.024* | 4.2 | 4.4 | 4.4 | 104% | <0.001** | 4.3 | 4.4 | 4.4 | 101% | 0.14 |  |
| Total Fat (g) | 14.6 | 14.7 | 15.6 | 107% | <0.001** | 14.7 | 15.1 | 15.1 | 102% | 0.06 | 14.7 | 15.1 | 15.1 | 102% | 0.006* |  |
| Saturated fat (g) | 6.7 | 6.8 | 7.3 | 109% | <0.001** | 6.8 | 7.1 | 7.0 | 103% | 0.12 | 6.8 | 7.1 | 7.0 | 102% | <0.001** |  |
| Sodium / Na (mg) | 351.0 | 356.4 | 377.1 | 107% | <0.001** | 355.5 | 366.6 | 363.2 | 102% | <0.001** | 356.5 | 365.9 | 363.9 | 102% | 0.046* |  |

*^1^ Adjusted for sex, age (continuous), physical activity (MET-min per week, continuous, imputed), measured height.*

*^2^ Differences between T1 and T3 were assessed using Wald tests on multiple quantile regression coefficients (adjustment for sex, age, physical activity and height, * P ≤ 0.05, ** P ≤ 0.001).*
