# Supplementary material for: Association between breakfast composition and abdominal obesity in the Swiss adult population eating breakfast regularly
Source: Int J Behav Nutr Phys Act. 2018 Nov 20;15:115. doi: 10.1186/s12966-018-0752-7 (PMC6247634; doi:10.1186/s12966-018-0752-7)
Supplement: Supplementary file 9 — Assessment of differences in nutrient intakes for the rest of the day by breakfast type. (DOCX 25 kb) [file 12966_2018_752_MOESM9_ESM.docx]

Additional file 9. Differences in nutrient intakes for the rest of the day (excluding breakfast intakes, unadjusted plus adjusted medians of the mean intake out of two 24-hour dietary recalls) by breakfast type (Tertiles, T3 vs. T1, N=1351).

Unadjusted medians:

|  | ‘Traditional’ – Pattern 1 | | | | | ‘Prudent’ – Pattern 2 | | | | | ‘Western’ – Pattern 3 | | | | | |
| --- | --- | --- | --- | --- | --- | --- | --- | --- | --- | --- | --- | --- | --- | --- | --- | --- |
|  | **T1** | **T2** | **T3** | **T3 / T1 (%)** | **P-Value** ^1^ | **T1** | **T2** | **T3** | **T3 / T1 (%)** | **P-Value** ^1^ | **T1** | **T2** | **T3** | **T3 / T1 (%)** | **P-Value** ^1^ |  |
| Energy (kcal) | 1625.1 | 1583.8 | 1731.7 | 107% | 0.018* | 1660.5 | 1647.1 | 1625.3 | 98% | 0.44 | 1581.0 | 1634.6 | 1718.6 | 109% | 0.004* |  |
| Protein (g) | 64.4 | 61.6 | 62.6 | 97% | 0.34 | 64.4 | 63.6 | 61.6 | 96% | 0.18 | 61.4 | 62.5 | 64.6 | 105% | 0.10 |  |
| Total carbohydrate (g) | 154.2 | 153.7 | 172.9 | 112% | <0.001** | 163.8 | 161.2 | 154.0 | 94% | 0.06 | 148.0 | 156.1 | 173.6 | 117% | <0.001** |  |
| Sugars (g) | 64.5 | 65.3 | 72.4 | 112% | 0.005* | 66.8 | 69.5 | 68.1 | 102% | 0.66 | 61.3 | 67.8 | 73.8 | 120% | <0.001** |  |
| Fiber (g) | 15.7 | 14.9 | 15.6 | 99% | 0.92 | 14.4 | 15.5 | 16.7 | 115% | <0.001** | 15.6 | 15.2 | 15.5 | 99% | 0.88 |  |
| Total Fat (g) | 67.2 | 66.6 | 69.9 | 104% | 0.28 | 68.0 | 67.6 | 67.9 | 100% | 0.99 | 66.1 | 67.5 | 71.4 | 108% | 0.019* |  |
| Saturated fat (g) | 23.0 | 23.0 | 25.7 | 112% | 0.002* | 23.7 | 24.8 | 24.0 | 101% | 0.73 | 23.3 | 23.9 | 25.1 | 108% | 0.046* |  |
| Alcohol (g) | 2.7 | 4.7 | 7.5 | 280% | <0.001** | 2.5 | 5.4 | 5.3 | 214% | 0.06 | 6.1 | 5.1 | 4.0 | 67% | 0.22 |  |
| Sodium / Na (mg) | 2242.5 | 2209.2 | 2512.7 | 112% | 0.001** | 2349.9 | 2331.8 | 2204.2 | 94% | 0.09 | 2220.5 | 2264.3 | 2420.1 | 109% | 0.027* |  |

*^1^ Differences between T1 and T3 were assessed using Wald tests on quantile regression coefficients (no adjustment, * P ≤ 0.05, ** P ≤ 0.001).*

Adjusted^1^ medians:

|  | ‘Traditional’ – Pattern 1 | | | | | ‘Prudent’ – Pattern 2 | | | | | ‘Western’ – Pattern 3 | | | | | |
| --- | --- | --- | --- | --- | --- | --- | --- | --- | --- | --- | --- | --- | --- | --- | --- | --- |
|  | **T1** | **T2** | **T3** | **T3 / T1 (%)** | **P-Value** ^2^ | **T1** | **T2** | **T3** | **T3 / T1 (%)** | **P-Value** ^2^ | **T1** | **T2** | **T3** | **T3 / T1 (%)** | **P-Value** ^2^ |  |
| Energy (kcal) | 1658.0 | 1637.2 | 1694.5 | 102% | 0.22 | 1686.0 | 1654.7 | 1647.9 | 98% | 0.73 | 1625.6 | 1655.1 | 1709.5 | 105% | 0.08 |  |
| Protein (g) | 64.3 | 63.7 | 66.7 | 104% | 0.74 | 65.7 | 64.7 | 64.2 | 98% | 0.020* | 63.2 | 64.7 | 66.7 | 106% | 0.53 |  |
| Total carbohydrate (g) | 163.3 | 159.9 | 164.2 | 101% | 0.012* | 166.7 | 160.6 | 159.9 | 96% | 0.86 | 157.9 | 161.0 | 169.0 | 107% | 0.010* |  |
| Sugars (g) | 68.0 | 67.5 | 68.9 | 101% | 0.024* | 68.9 | 67.8 | 67.4 | 98% | 0.49 | 66.6 | 67.9 | 69.8 | 105% | <0.001** |  |
| Fiber (g) | 15.3 | 15.2 | 15.5 | 101% | 0.45 | 15.4 | 15.3 | 15.3 | 99.7% | <0.001** | 15.2 | 15.3 | 15.5 | 102% | 0.35 |  |
| Total Fat (g) | 68.9 | 68.2 | 70.1 | 102% | 0.31 | 69.5 | 68.7 | 68.7 | 99% | 0.19 | 67.7 | 68.7 | 70.5 | 104% | 0.21 |  |
| Saturated fat (g) | 24.5 | 24.4 | 25.3 | 103% | 0.07 | 24.7 | 24.7 | 24.7 | 100.1% | 0.67 | 24.3 | 24.7 | 25.1 | 103% | 0.13 |  |
| Alcohol (g) | 5.3 | 5.7 | 7.2 | 136% | 0.69 | 5.6 | 6.4 | 6.2 | 110% | 0.90 | 5.8 | 6.4 | 6.1 | 104% | 0.44 |  |
| Sodium / Na (mg) | 2341.8 | 2304.1 | 2377.6 | 102% | 0.22 | 2398.2 | 2318.8 | 2298.6 | 96% | 0.50 | 2281.0 | 2320.6 | 2415.7 | 106% | 0.17 |  |

*^1^ Adjusted for sex, age (continuous), physical activity (MET-min per week, continuous, imputed), measured height.*

*^2^ Differences between T1 and T3 were assessed using Wald tests on multiple quantile regression coefficients (adjustment for sex, age, physical activity and height, * P ≤ 0.05, ** P ≤ 0.001).*
