# Supplementary material for: Association between breakfast composition and abdominal obesity in the Swiss adult population eating breakfast regularly
Source: Int J Behav Nutr Phys Act. 2018 Nov 20;15:115. doi: 10.1186/s12966-018-0752-7 (PMC6247634; doi:10.1186/s12966-018-0752-7)
Supplement: Supplementary file 10 — Assessment of differences in food intakes for the rest of the day by breakfast ype. (DOCX 25 kb) [file 12966_2018_752_MOESM10_ESM.docx]

Additional file 10. Differences in food group intakes for the rest of the day (excluding breakfast intakes, mean score from 0 to 10 per component, derived from the mean intake out of two 24-hour dietary recalls) by breakfast type (Tertiles, T3 vs. T1, N=1351).

*See Additional file 5 for the calculation of the nutritional score*

Unadjusted means:

|  |  | ‘Traditional’ – Pattern 1 | | | |  | ‘Prudent’ – Pattern 2 | | | |  | | ‘Western’ – Pattern 3 | | | | |
| --- | --- | --- | --- | --- | --- | --- | --- | --- | --- | --- | --- | --- | --- | --- | --- | --- | --- |
|  | **T1** | **T2** | **T3** | **T3 / T1 (%)** |  | **T1** | **T2** | **T3** | **T3 / T1 (%)** | **P-Value**^1^ | **T1** | **T2** | | **T3** | **T3 / T1 (%)** | **P-Value** ^1^ |  |
| Vegetables | 3.4 | 3.4 | 3.2 | 93% | 0.08 | 3.1 | 3.2 | 3.7 | 121% | <0.001** | 3.6 | 3.3 | | 3.1 | 85% | <0.001** |  |
| Fruit | 3.2 | 3.2 | 3.1 | 97% | 0.59 | 2.7 | 3.3 | 3.5 | 129% | <0.001** | 3.2 | 3.2 | | 3.1 | 96% | 0.53 |  |
| Whole grain | 2.6 | 2.2 | 2.0 | 76% | 0.003* | 2.0 | 2.1 | 2.8 | 145% | <0.001** | 2.5 | 2.1 | | 2.3 | 92% | 0.38 |  |
| Sugary drinks | 6.5 | 6.2 | 5.7 | 88% | 0.008* | 5.7 | 6.0 | 6.8 | 119% | <0.001** | 6.6 | 6.1 | | 5.7 | 87% | 0.003* |  |
| Nuts and legumes | 2.2 | 1.9 | 1.7 | 77% | 0.030* | 1.6 | 1.7 | 2.5 | 157% | <0.001** | 2.3 | 1.9 | | 1.6 | 71% | 0.003* |  |
| Meat | 4.9 | 4.3 | 4.2 | 85% | 0.004* | 3.7 | 4.3 | 5.4 | 147% | <0.001** | 4.9 | 4.3 | | 4.1 | 83% | <0.001** |  |
| Total score | 22.9 | 21.3 | 19.9 | 87% | <0.001** | 18.8 | 20.5 | 24.8 | 132% | <0.001** | 23.1 | 21.1 | | 19.8 | 86% | <0.001** |  |

*^1^ Differences between T1 and T3 were assessed using Wald tests on simple linear regression coefficients (no adjustment, * P ≤ 0.05, ** P ≤ 0.001).*

Adjusted^1^ means:

|  |  | ‘Traditional’ – Pattern 1 | | | |  | ‘Prudent’ – Pattern 2 | | | |  | | ‘Western’ – Pattern 3 | | | | |
| --- | --- | --- | --- | --- | --- | --- | --- | --- | --- | --- | --- | --- | --- | --- | --- | --- | --- |
|  | **T1** | **T2** | **T3** | **T3 / T1 (%)** | **P-Value** ^2^ | **T1** | **T2** | **T3** | **T3 / T1 (%)** | **P-Value** ^2^ | **T1** | **T2** | | **T3** | **T3 / T1 (%)** | **P-Value** ^2^ |  |
| Vegetables | 3.3 | 3.3 | 3.4 | 101% | 0.033* | 3.3 | 3.4 | 3.4 | 103% | <0.001** | 3.4 | 3.4 | | 3.3 | 98% | <0.001** |  |
| Fruit | 3.1 | 3.2 | 3.2 | 104% | 0.22 | 3.1 | 3.2 | 3.2 | 105% | <0.001** | 3.2 | 3.2 | | 3.1 | 96% | 0.97 |  |
| Whole grain | 2.3 | 2.3 | 2.3 | 97% | 0.006* | 2.3 | 2.3 | 2.3 | 99% | <0.001** | 2.3 | 2.3 | | 2.3 | 102% | 0.23 |  |
| Sugary drinks | 6.1 | 6.2 | 6.1 | 101% | 0.002* | 5.9 | 6.2 | 6.3 | 106% | 0.008* | 6.3 | 6.2 | | 5.9 | 93% | 0.12 |  |
| Nuts and legumes | 2.0 | 2.0 | 1.9 | 93% | 0.12 | 2.0 | 1.9 | 1.9 | 99% | <0.001** | 2.0 | 1.9 | | 1.9 | 100% | 0.003* |  |
| Meat | 4.6 | 4.6 | 4.3 | 94% | 0.06 | 4.4 | 4.5 | 4.5 | 102% | <0.001** | 4.6 | 4.5 | | 4.3 | 95% | 0.014* |  |
| Total score | 21.4 | 21.5 | 21.1 | 99% | <0.001** | 20.9 | 21.4 | 21.6 | 103% | <0.001** | 21.7 | 21.4 | | 20.9 | 96% | <0.001** |  |

*^1^ Adjusted for sex, age (continuous), physical activity (MET-min per week, continuous, imputed), measured height.*

*^2^ Differences between T1 and T3 were assessed using Wald tests on multiple linear regression coefficients (adjustment for sex, age, physical activity and height, * P ≤ 0.05, ** P ≤ 0.001).*
