# Supplementary material for: Association between breakfast composition and abdominal obesity in the Swiss adult population eating breakfast regularly
Source: Int J Behav Nutr Phys Act. 2018 Nov 20;15:115. doi: 10.1186/s12966-018-0752-7 (PMC6247634; doi:10.1186/s12966-018-0752-7)
Supplement: Supplementary file 11 — Association between breakfast type and WHR (continuous). (DOCX 21 kb) [file 12966_2018_752_MOESM11_ESM.docx]

Additional file 11. Association between ‘prudent’ breakfast and abdominal obesity (based on log WHR, continuous, N=1351).

|  | **‘Prudent’ – Fruit, unprocessed and unsweetened cereal flakes, nuts/seeds and yogurt** | | | | | | | |
| --- | --- | --- | --- | --- | --- | --- | --- | --- |
|  | **T1** | **T2** | | | **T3** | | |  |
|  | **β** | **β** | **95%** **CI** | | **β** | **95% CI** | | **P-Value for trend**^3^ |
| **Crude** | 0 (ref) | 0.0211 | 0.0064 | 0.0359 | 0.0000 | -0.0147 | 0.0147 | 0.99 |
| **Model 1**  *(sex + age)* | 0 (ref) | 0.0004 | -0.0092 | 0.0100 | -0.0154 | -0.0250 | -0.0058 | 0.002* |
| **Model 2**  *(age + sex + physical activity + total energy intake)^1^* | 0 (ref) | 0.0012 | -0.0084 | 0.0109 | -0.0153 | -0.0249 | -0.0056 | 0.002* |
| **Model 3**  *(11 covariates)^1^* | 0 (ref) | 0.0007 | -0.0097 | 0.0098 | -0.0155 | -0.0255 | -0.0054 | 0.002* |
| **Model 4**  *(16 covariates, including diet quality during the rest of the day – nutrient + food-based approach)^2^* | 0 (ref) | 0.0015 | -0.0083 | 0.0113 | -0.0120 | -0.0222 | -0.0018 | 0.020* |

*^1^ Sex, age (continuous), physical activity (MET-min per week, continuous, imputed), total energy intake (mean out of two 24-hour dietary recalls), alcohol intake (mean intake out of two 24-hour dietary recalls), education (university degree: yes / no), food literacy (knowing about the Swiss Food Pyramid: yes / no), smoking (never / past / current), nationality (Swiss / non-Swiss), household status (alone / couple with children / couple without children), season of the first 24-hour dietary recall (cold / warm), linguistic region (German / French / Italian).*

*^2^ Idem plus diet quality during the rest of the day (outside breakfast) considering dietary fiber, saturated fat, sodium, and the six-food-component nutritional score (mean intake out of two 24-hour dietary recalls).*

*^3^ Differences were assessed using multiple logistic regressions (* P ≤ 0.05).*
