# Supplementary material for: Association between breakfast composition and abdominal obesity in the Swiss adult population eating breakfast regularly
Source: Int J Behav Nutr Phys Act. 2018 Nov 20;15:115. doi: 10.1186/s12966-018-0752-7 (PMC6247634; doi:10.1186/s12966-018-0752-7)
Supplement: Supplementary file 12 — Association between the ‘prudent’ breakfast and four obesity anthropometric parameters. (DOCX 104 kb) [file 12966_2018_752_MOESM12_ESM.docx]

Additional file 12. Association between the three breakfast types and four obesity anthropometric parameters.

*
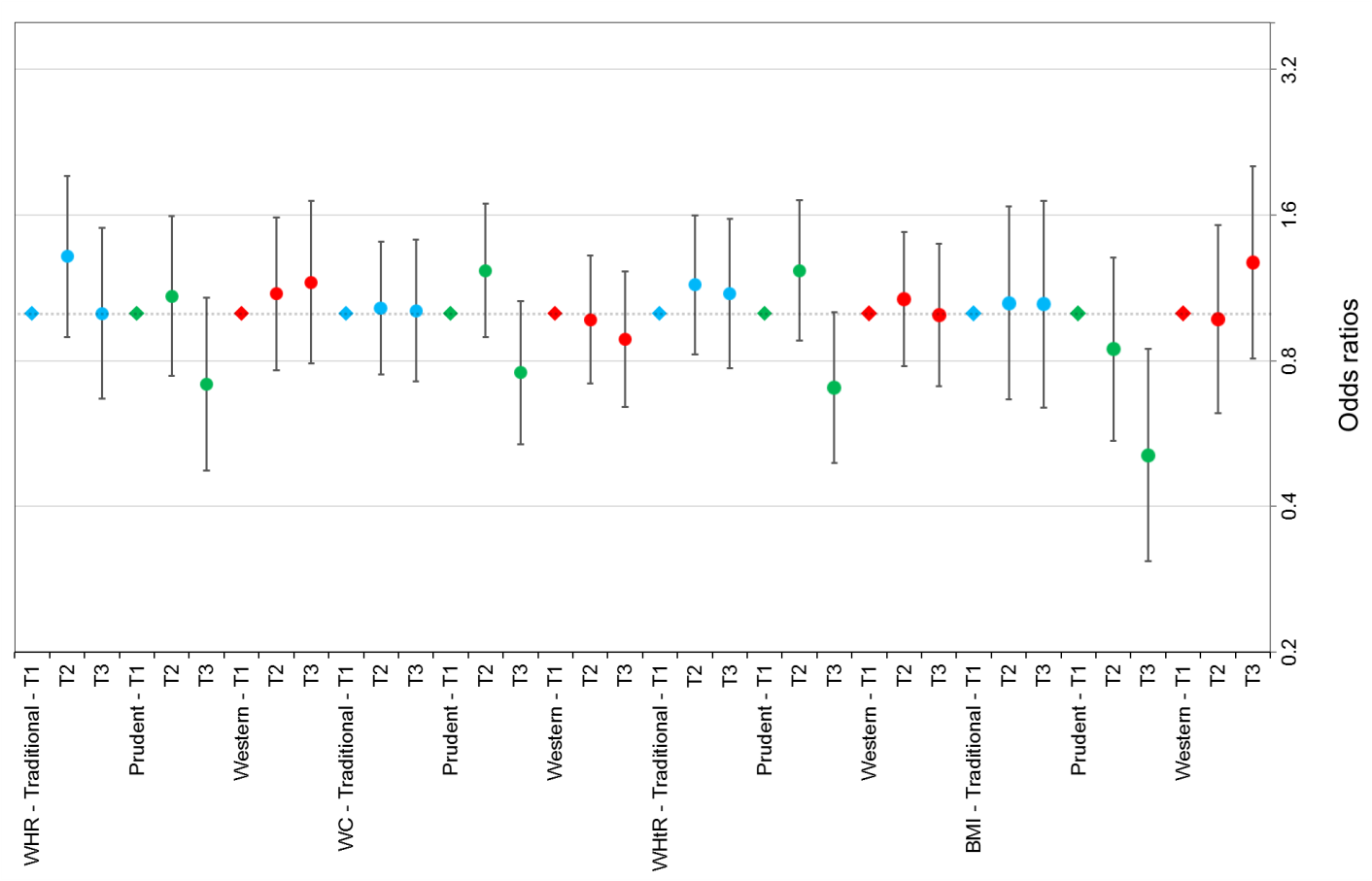
*

*Odds ratios between the three breakfast types (Tertiles 1 to 3: T3 being closely associated with the pattern) and abdominal obesity (waist-to-hip ratio (WHR): ≥ 0.9 (♂); ≥ 0.85 (♀); waist circumference (WC): > 90 cm (♂); > 84 cm (♀), waist-to-height ratio (WHtR): ≥ 0.5 (♂, ♀), body mass index (BMI): ≥ 30 kg/m2 (♂, ♀), N=1 351). The logistic models were adjusted for sex, age, physical activity, total energy intake, alcohol intake, education, food literacy, smoking, nationality, household status, season of the first 24-hour dietary recall, linguistic region, and diet quality during the rest of the day (outside breakfast).*
