# Supplementary material for: Association between breakfast composition and abdominal obesity in the Swiss adult population eating breakfast regularly
Source: Int J Behav Nutr Phys Act. 2018 Nov 20;15:115. doi: 10.1186/s12966-018-0752-7 (PMC6247634; doi:10.1186/s12966-018-0752-7)
Supplement: Supplementary file 13 — Distribution of regular breakfast eaters in the three breakfast types. (DOCX 20 kb) [file 12966_2018_752_MOESM13_ESM.docx]

Additional file 13. Distribution of regular breakfast eaters (N=1351) in the three breakfast types (Tertile 3, T3, is the one closely associated with the pattern).
